# Glucose transporters in pancreatic islets

**DOI:** 10.1007/s00424-020-02383-4

**Published:** 2020-05-12

**Authors:** Constantin Berger, Daniela Zdzieblo

**Affiliations:** 1 Tissue Engineering & Regenerative Medicine, University Hospital Würzburg, Röntgenring 11, 97070 Würzburg, Germany; 2 Fraunhofer Institute for Silicate Research (ISC), Translational Center Regenerative Therapies, Neunerplatz 2, 97082 Würzburg, Germany

**Keywords:** Glucose transport, Pancreatic islet, β-Cell, α-Cell, GLUTs, SGLTs

## Abstract

The fine-tuning of glucose uptake mechanisms is rendered by various glucose transporters with distinct transport characteristics. In the pancreatic islet, facilitative diffusion glucose transporters (GLUTs), and sodium-glucose cotransporters (SGLTs) contribute to glucose uptake and represent important components in the glucose-stimulated hormone release from endocrine cells, therefore playing a crucial role in blood glucose homeostasis. This review summarizes the current knowledge about cell type-specific expression profiles as well as proven and putative functions of distinct GLUT and SGLT family members in the human and rodent pancreatic islet and further discusses their possible involvement in onset and progression of *diabetes mellitus*. In context of GLUTs, we focus on GLUT2, characterizing the main glucose transporter in insulin-secreting β-cells in rodents. In addition, we discuss recent data proposing that other GLUT family members, namely GLUT1 and GLUT3, render this task in humans. Finally, we summarize latest information about SGLT1 and SGLT2 as representatives of the SGLT family that have been reported to be expressed predominantly in the α-cell population with a suggested functional role in the regulation of glucagon release.

## Introduction

Glucose is a main energy source for many organisms and covers a central role in cell metabolism. However, the way in which glucose is transported across the cell membrane is not a uniform process. Various mechanisms of glucose uptake are found in different tissues, thereby meeting the energetic and functional requirements of different cell types. The regulation of glucose uptake is rendered by the cell type-specific expression of distinct glucose transporters with individual transport characteristics. Understanding the expression, regulation and function of different glucose transporters as well as their possible crosstalk is key for unraveling biological and pathological glucose-dependent processes.

Controlled glucose uptake and metabolism is of outmost importance for the sustained function of the endocrine pancreas, which is composed of five different cell types forming the islets of Langerhans. By secretion of hormones that mediate changes in the glucose metabolism of peripheral cells, the endocrine pancreas is able to maintain blood glucose levels at a physiological range. Loss of glycemic control due to an impaired hormone secretion or an increased hormone insensitivity of peripheral cells can lead to hyperglycemia, a pathological condition diagnosed as *diabetes mellitus*.

Blood glucose homeostasis is predominantly regulated by the antagonistic interplay of the endocrine α- and β-cells. While α-cells respond to hypoglycemia with the release of glucagon resulting in the elevation of circulating glucose, β-cells secrete insulin at hyperglycemic conditions leading to the reduction of plasma glucose levels. The ability of α- and β-cells to regulate plasma glucose levels in the body is tightly linked to their ability in detecting changes in the extracellular glucose levels. In order to fulfill this function, endocrine cells require specialized glucose transport mechanisms allowing for a continuous monitoring of extracellular glucose concentrations and a rapid adaption of hormone secretion.

In the pancreatic endocrine cells glucose uptake is particularly rendered by members of the facilitative glucose transporter (GLUT) family (*SLC2*) and the sodium-glucose cotransporter (SGLT) family (*SLC5*) that both belong to the 52 families comprising solute carrier (*SLC*) superfamily [6, 24, 51, 88, 162–164]. In this review, we summarize the current knowledge about GLUTs and SGLTs in the pancreatic islet and their intended roles in health and disease.

## GLUTs in the pancreas

GLUTs facilitate the passive transport of glucose across the plasma membrane [88]. In humans, members of the GLUT family are encoded by 14 different *SLC2* genes and subdivided according to their substrate specificities [5, 51]. Class I comprises GLUT1–4 (*SLC2A1*–*4*) and GLUT14 (*SLC2A14*) that transport glucose with diverse affinities ranging from low to high [5]. Class II encompasses GLUT5, 7, 9, and 11 (*SLC2A5*, *7*, *9*, and *11*) that primarily mediate the uptake of fructose [5]. Class III includes the glucose transporters GLUT6, 8, 10, 12 and 13 (*SLC2A6*, *8*, *10*, *12*, and *13*) as well as the H^+^/myoinositol transporter HMIT (GLUT13; *SLC2A13*) [5]. Most of our current knowledge about GLUTs in the pancreas originates from studies focusing on β-cells and their role in glucose-induced insulin release. In contrast to that, surprisingly little is known about the role of GLUTs in other cell types of the islet. Recent gene expression studies reveal specific *SLC2* expression patterns in all cell types of the islet and indicate a diversity in GLUT-mediated glucose uptake (Table 1).

**Table 1:** Overview on documented SLC2 gene expression in human islet cells

	Cell type							
Gene	α	β	γ	δ	ε	acinar cells	duct cells	References
SLC2A1	x	x	x	x	x	x	x	[48, 82, 114, 128, 133]
SLC2A2	–	x	–	–	–	–	–	[38, 48, 82, 128, 133]
SLC2A3	x	x	x	x	x	–	–	[48, 82, 114, 133]
SLC2A4	–	–	–	–	–	–	–	[133]
SLC2A5	–	–	–	–	–	–	–	[133]
SLC2A6	x	x	x	x	x	x	x	[133]
SLC2A7	–	–	–	–	–	–	–	[133]
SLC2A8	x	x	x	x	–	x	x	[133]
SLC2A9	–	x	–	–	–	x	x	[31, 133]
SLC2A10	–	–	–	–	–	x	x	[133]
SLC2A11	x	x	x	x	x	–	x	[133]
SLC2A12	x	x	x	x	x	x	x	[133]
SLC2A13	x	x	x	x	x	x	x	[133]
SLC2A14	–	–	–	–	–	x	–	[133]

### Role of GLUTs in the regulation of hormone release by α- and β-cells

**Fig. 1:**
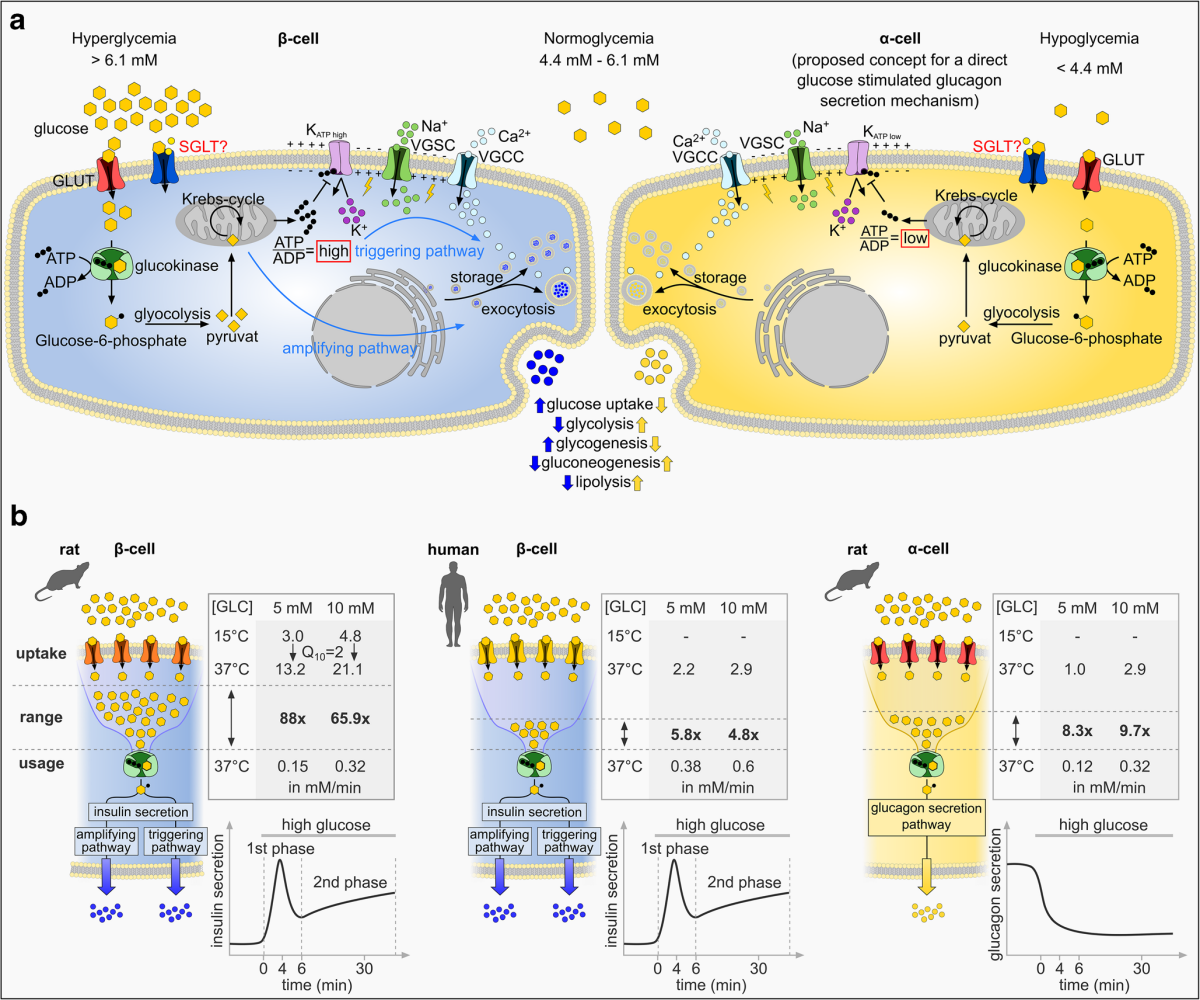
Physiology of the human and rodent β- and α-cell. **a** Insulin secretion from β-cells (blue) is triggered at high glucose concentrations. Glucose enters the cell mainly via GLUTs. The contribution of SGLT to glucose uptake in β-cells is not yet confirmed. Intracellularly, glucose is phosphorylated by the glucokinase and converted to pyruvate during glycolysis. Pyruvate enters the mitochondria where it is metabolized in the Krebs cycle, resulting in the generation of ATP. Elevation in the ATP/ADP ratio induces the closure of ATP-sensitive potassium channels (K_ATP_) leading to the depolarization of the cell membrane. Opening of voltage-gated sodium channels (VGSC) causes a further depolarization by the influx of sodium ions (Na^+^) causing an influx of calcium ions (Ca^2+^) via voltage-gated calcium channels (VGGC). Elevation of the intracellular Ca^2+^-concentration [Ca^2+^]_i_ triggers the exocytosis of insulin-filled vesicles by which insulin is released. Insulin secretion is mediated by the triggering pathway, which includes the rapid increase of [Ca^2+^]i resulting in a fast insulin response, as well as by the metabolic amplifying pathway that generates a second, continous release of insulin. Glucagon secretion from α-cells is less well understood. Of the several theories, which are currently discussed, one possible mechanism is illustrated in the displayed α-cell (yellow). According to this idea, α-cells share many features of the β-cells including uptake glucose via GLUTs and presumably SGLT1. Contrary to β-cells, K_ATP_s are thought to close already at a low ATP/ADP ratio resulting in glucagon secretion, whereas a further increase of intracellular ATP induces the closure of VGSCs and VGGCs, thereby inhibiting glucagon release. Insulin and glucagon have opposing effects on peripheral cells and mediate signals for a reduction or rise of plasma glucose levels. **b** Analysis of glucose transport of human and rat β- and rat α-cells measured by the uptake of 3-O-Methyl-d-glucose (3-OMG).  In the rat β-cell (left) glucose uptake via specific glucose carriers (orange) is faster than the subsequent glucose phosphorylation by glucokinase. The activation of the triggering pathway as well as the metabolic amplifying pathway results in a biphasic insulin secretion in response to a rapid increase of glucose concentrations. Human β-cells (middle) exhibit a different subset of glucose transporters (yellow) that presumably results in a slower glucose uptake and a smaller gap between glucose uptake and usage compared to rat β-cells. Glucose uptake rate in rat α-cells (right) is comparable to human β-cells and similarly mediated by a specific subset of glucose transporters (red). Glucose-stimulated glucagon secretion decreases at high glucose concentration

GLUT-mediated glucose uptake represents an essential element in the glucose-dependent insulin secretion pathway of β-cells (Fig. 1a). Upon uptake via GLUTs, glucose is phosphorylated by glucokinase and metabolized to pyruvate during glycolysis. Mitochondrial oxidation of pyruvate in the tricarboxylic cycle results in an accumulation of intracellular ATP, which triggers depolarization of the plasma membrane by closure of ATP-dependent potassium (K^+^) channels (K_ATP_). As a consequence voltage-gated calcium (Ca^2+^) channels (VGCCs) open, leading to an increase in cytoplasmic Ca^2+^ ([Ca^2+^]_i_), which induces exocytosis of insulin-containing vesicles. The process of glucose-stimulated insulin secretion (GSIS) proceeds in a biphasic manner. Initially, large amounts of insulin are secreted shortly after the rise of extracellular glucose, which is followed by a sustained insulin exocytosis above basal secretion levels.

In rodent β-cells, glucose uptake is mediated by the low affinity glucose transporter GLUT2 (Km ~ 15–20 mmol/l) [144]. Its high transport capacity facilitates the equilibration between extracellular and intracellular glucose levels and allows β-cells to rapidly adapt to rising glucose concentrations [7]. The subsequent phosphorylation by glucokinase (Km ~ 4–10 mmol/l) proceeds in a much slower rate than GLUT2-mediated uptake [84, 113]. Studies investigating the glucose turnover in freshly isolated rat islets using radiometric measurements based on the liberation of ^3^H_2_O from (5-3H)-glucose demonstrated that glucose utilization by the glucokinase (0.29 mmol/l/min) was about 100 times slower than the calculated glucose uptake (28.96 mmol/l/min) [75, 142]. Heimberg et al. made a similar observation in β-cells purified from isolated rat islets [52]. Glucose uptake was measured at 15 °C to allow for accurate calculation using the non-metabolizable glucose analog 3-O-Methyl-d-glucose (3-OMG) and glucose utilization experiments were performed at 37 °C using (5-3H)-glucose. The experiments revealed a glucose uptake of 0.7, 3.0, and 4.8 mmol/l and a glucose utilization of 0.04, 0.15, and 0.32 mmol/l/min in 1, 5, and 10 mmol/l glucose, respectively [52]. Assuming a Q_10_ value of 2 to consider the differing experimental temperatures, glucose uptake in rat β-cells was calculated to be 60–90 times higher than glucose utilization by glucokinase [52] (Fig. 1b). Accordingly, it is not the GLUT2-mediated glucose uptake, but the glucokinase phosphorylation that is the rate-limiting factor for glucose utilization and GSIS in rodent β-cells [28, 41, 133, 142].

Although not being the rate-limiting step, GLUT2-mediated glucose transport is essential for insulin secretion of rodent β-cells. Studies on GLUT2 knockout (*Slc2a2*^*−/−*^*)* mice exhibited a diminished glucose clearance and insulin plasma levels as a result of an impaired GSIS [46]. Pancreases isolated from these mice lacked an appropriate GSIS, while the insulin release in response to glucose metabolites, such as D-glyceraldehyde was retained, proving that the impaired GSIS is the result of a reduced intracellular glucose concentration [46, 47]. Islets isolated from GLUT2 knockout mice showed a slight increase in glucose utilization at glucose concentrations between 1 and 6 mmol/l but no further elevation between 6 and 20 mmol/l glucose demonstrating the requirement of GLUT2 function for the intracellular glucose equilibration at high glucose levels in mouse β-cells [46, 47]. Interestingly, ectopic expression of the low affinity transporter GLUT1 (Km ~ 1–5) in GLUT2-deficient mice restores GSIS, proving that under normal conditions, the mechanism of glucose entry into the cell is not decisive for sustained β-cell function as long as glucose transport exceeds glucose metabolism [147].

GLUT2-deficient mice as well as islets isolated from these mice lacked a fast insulin response to hyperglycemic stimuli but retained a second-phase of insulin secretion and a reduced increase of GSIS to elevations of glucose from 6 to 20 mmol/l [46, 47]. It is known that the first phase of insulin secretion is mainly induced by a rapid increase of [Ca^2+^] in the course of the triggering pathway. In contrast, the second phase of insulin secretion presumably originates from a metabolic amplifying pathway, which augments the [Ca^2+^]-mediated effects of the first phase (see [54] for further information). In mice, K_ATP_-channels close at an intracellular glucose concentration of 6–7 mmol/l resulting in depolarization and induction of the triggering pathway [53]. GLUT2-deficient mice obviously lacked activation of the triggering pathway suggesting that glucose uptake in mice lacking GLUT2 is not sufficient to reach the required threshold [46]. The retained second phase insulin secretion argue for an additional GLUT2-indepent glucose uptake that allows for reduced GSIS without activating the triggering pathway. Studies with rats showed that a “slow ramp” increase of glucose concentration results in a gradual rise in insulin secretion without a first phase [45, 138]. The assumption of a slow glucose uptake as an explanation for retained second phase insulin secretion hypothesizes the existence of a low Km high affinity transporter in mice. Guillam et al. demonstrated the presence of the high affinity transporter GLUT1 in mouse islets, but could not allocate its expression to a certain cell type due to its low abundance [47]. Another candidate is the high-affinity transporter GLUT9 (Km ~ 0.6) [31]. Both splice forms of the *Slc2a9* gene (GLUT9a and GLUT9b) were found in murine β-cells, whereas only GLUT9b showed a plasma membrane localization. So far, functional studies were only conducted with Min-6 as well as the rat insulinoma INS cells. In both cell lines RNAi-induced knockdown of GLUT9 resulted in reduced intracellular ATP levels and a diminished GSIS in presence of GLUT2 [31]. To verify the potential involvement of GLUT9 in β-cell GSIS, in vitro experiments with isolated islets and in vivo studies are required.

Contrary to β-cells, the precise cellular mechanisms underlying glucagon secretion remain less understood. Current concepts comprise indirect and direct glucose signaling mechanisms (Fig. 1a). Recently, the involvement of SGLTs in the glucose transport of α-cells has generated huge interest, which is reviewed in detail in the following chapters.

In terms of GLUT expression and function in pancreatic α-cells, limited data is available [52, 57, 117, 133]. An early study on rat islets suggested that GLUT1 is the main GLUT transporter in rat α-cells [52]. According to this study, GLUT1 was more abundant in rat α-cells compared to β-cells, while both cell types exhibited a similar glucokinase expression and function [52, 113]. Heimberg et al. further demonstrated that glucose uptake in rat α-cells (0.12 and 0.32 mmol/l/min) by GLUT1 was one magnitude lower compared to rat β-cells (13.2 and 21.1 mmol/l/min) at glucose concentrations of 5 and 10 mmol/l, respectively, whereas no difference in glucose utilization was detected [52]. Despite a slower uptake compared to β-cells, GLUT1-mediated glucose transport in α-cells was still 8–9 times higher than glucose utilization, indicating that also in α-cells glucokinase is the rate-limiting step for glycolysis [52] (Fig. 1b). This finding suggests that GLUT1 in rodent α-cells might fulfill a similar function as GLUT2 does in rodent β-cells. Blocking of GLUT1 with phloretin resulted in an increase in glucagon secretion, emphasizing the idea that GLUT1 mediates a direct glucose-dependent effect on glucagon secretion [141].

### Human β-cells do not rely on GLUT2

**Fig. 2:**
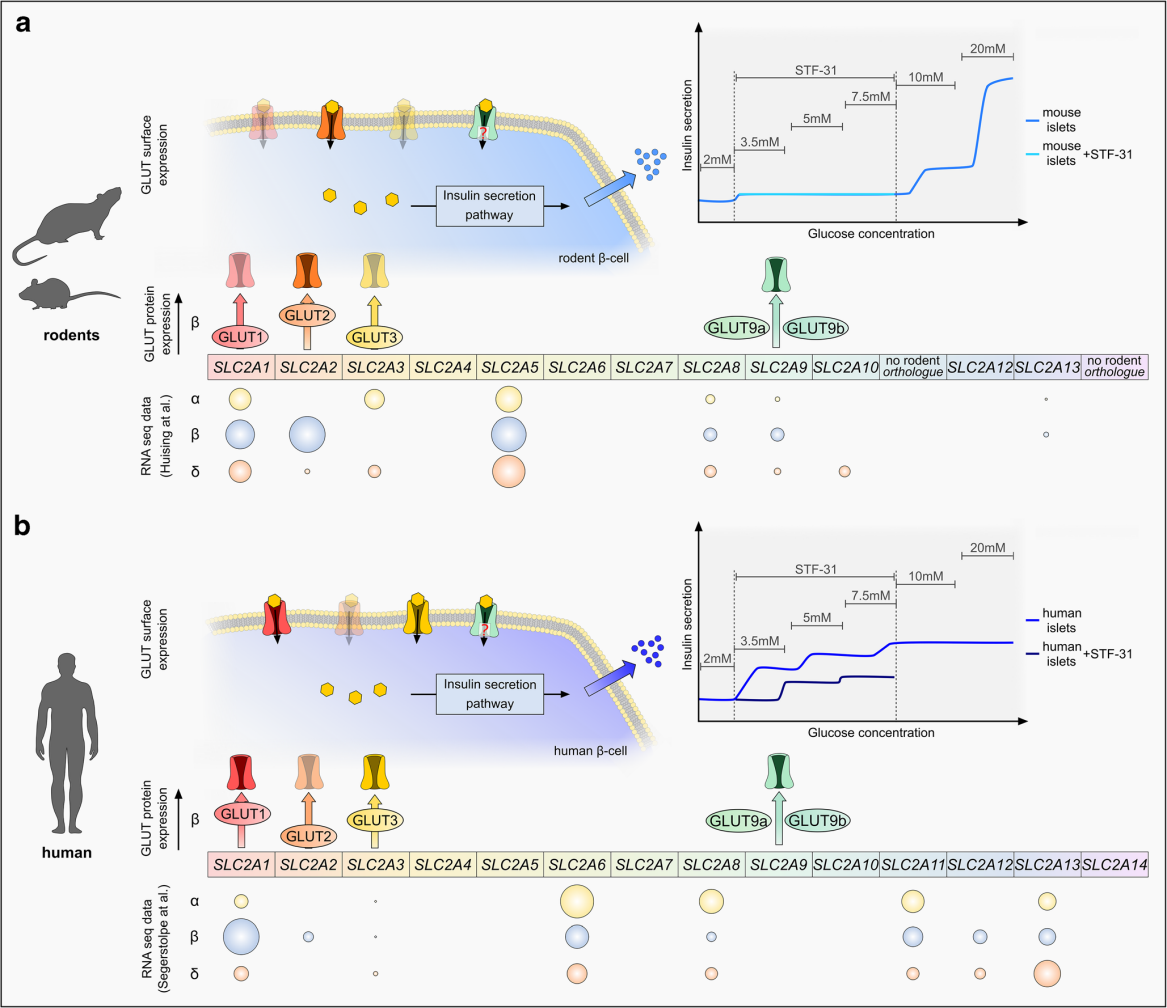
Comparison of GLUT mRNA and protein expression in rodent and human islet cells. **a** Overview of *Slc2* gene and proposed GLUT protein expression in rodents. RNA seq data: gene expression of murine *Slc2* genes was determined by analyzing RNA sequencing data of murine FACS-sorted α-, β-, and δ-cells published by DiGruccio et al. [25]. Gene expression is shown as spheres representing the natural log of the normalized expression values (ln(RPKM)). Sphere size corresponds to the mRNA abundance of each gene normalized to the highest value of the analyzed dataset. GLUT protein expression: schematic representation of documented GLUT protein expression in murine β-cells. The position of the GLUTs along the arrow indicates the protein abundance estimated according to published studies. GLUT surface expression: schematic illustration of the proposed GLUT expression pattern in rodent β-cells, suggesting GLUT2 (orange) to cover a dominant role in glucose transport in comparison to GLUT1 (translucent red) and GLUT3 (translucent yellow). The contribution of GLUT9a and GLUT9b (green) remains speculative. Insulin release: Scheme of GSIS demonstrating the responsiveness of mouse islets to different glucose concentration. Mouse islets show an increased insulin secretion at high glucose concentration and show no reaction upon the addition of the selective GLUT1 inhibitor STF-31 [114]. **b** Overview of *SLC2* expression and proposed GLUT protein expression in humans. RNA seq data: gene expression of human *SLC2* genes was determined by analyzing single cell RNA sequencing data of human α-, β- and δ-cells published by Segerstolpe et al. [133]. (see also http://sandberg.cmb.ki.se/pancreas/). Spheres represent gene expression determined by the natural log of the normalized expression values (ln(RPKM)). Sphere size reflects the mRNA abundance of each gene normalized to the highest value of the analyzed dataset. GLUT protein expression: illustration of documented GLUT expression in the human β-cell. The position of the GLUTs along the arrow indicates the protein abundance estimated according to published studies. GLUT surface expression: schematic illustration of the proposed GLUT pattern contributing to glucose transport in human β-cells comprising mostly GLUT1 (red) and GLUT3 (yellow) as well as to a less extent GLUT2 (translucent orange). The contribution of GLUT9a and GLUT9b (green) is not proven. Insulin release: scheme of GSIS of human islets demonstrating a different responsiveness of human islets to increasing glucose levels compared to murine islets according to a study by Pingitore et al. [114]. Human islets react to the transient application of the selective GLUT1 inhibitor STF-31 with a reduced amplitude in insulin release

The role of GLUTs in human β-cells is controversial. Although GLUT2 is present in human β-cells at background levels, there is an increasing amount of evidence suggesting GLUT1 and GLUT3 to be the predominant glucose transporters in human β-cells [20, 82, 152]. Figure 2 summarizes the current understanding of GLUT expression in human islets and contrasts the known differences to their rodent counterparts.

Studies on the abundance of GLUT2 in human β-cells provide conflicting data. Several groups demonstrated GLUT2 expression in mature human islets or during early islet development, albeit at very low levels compared to rodent samples [38, 82, 88, 132]. In line with that, clinical studies identified defective GLUT2 as a cause for gestational and neonatal *diabetes*, suggesting that GLUT2 transiently presents as an integral part of human β-cell physiology [87, 124]. In contrast, De Vos et al. demonstrated GLUT2 to be absent in isolated human pancreatic islets and revealed a major interspecies discrepancy in comparison to rat islets, which express GLUT2 [152]. These findings are supported by a recent study that analyzed sets of 207 intact human islets from the “Translational Human Pancreatic Islet Genotype Tissue-Expression Resource” (TIGER) [33, 150] and found lower *SLC2A2* expression compared to *SLC2A1* [128]. In the course of this review, we further analyzed existing datasets from transcriptome studies of FACS purified mouse islet cells [25] (Fig. 2a) and single cell RNA sequencing analysis of human islets [133] (Fig. 2b) for *SLC2* gene expression. The sample sets reveal profound interspecies differences between rodents and humans in the overall *SLC2* expression pattern of endocrine cells. Moreover, the data confirm an increased expression of *SLC2A1* in human β-cells compared to *SLC2A2*, whereas murine human β-cells exhibit elevated *SLC2A2* expression.

Beside the conflicting expression data about GLUT2 there are hints arguing against an extensive participation of GLUT2 in glucose uptake in human β-cells. Firstly, human islets are resistant to streptozotocin (STZ), a β-cell toxin which is transported into the cell via GLUT2 [29, 30, 131, 156]. Secondly, in humans homozygous GLUT2 mutations manifest in postprandial hyperglycemia but symptoms are not comparable with the severe β-cell failure observed in the *Slc2a2*^*−/−*^ mouse model [135]. Thirdly, glucose uptake of human β-cells differs from murine β-cells. In a study measuring glucose transport of dissociated β-cells, uptake of 2-[N-(7-nitrobenz-2-oxa-1,3-diazol-4-yl)amino]-2-deoxy-D-glucose (2-NBDG), a fluorescent glucose analog at 10 mmol/l glucose, was lower in human β-cells (0.9 nmol/2 min/1 × 10^5^ cells) compared to murine β-cells (0.8 nmol/2 min/5 × 10^4^ cells) [101] indicating an alternative glucose transport mechanism in human β-cells.

While these evidences argue against a GLUT2-dependent glucose uptake in human β-cells, other GLUTs, namely GLUT1 and GLUT3 (Km ~ 1–2) are proposed to cover the role of main glucose transporters in human β-cells [20, 48, 152] (Fig. 2b). In human islets, GLUT1 and GLUT3 showed a dominant expression over GLUT2 on mRNA and protein level and were elevated in comparison to mouse islets [114, 152]. McCulloch et al. confirmed the predominant expression of GLUT1 and GLUT3 over GLUT2 in human islets and could allocate GLUT1 and GLUT3 expression to the plasma membrane of β-cells [82]. Another study further provided evidence that GLUT1 is uniformly dominant over GLUT3 in human β-cells suggesting GLUT1 to be the main glucose transporter in human insulin-secreting β-cells [20].

Studies on glucose transport in human islets support the idea of a predominant role of GLUT1 and GLUT3. De Vos et al. measured 3-OMG uptake in dissociated human islet cells at 5 and 10 mmol/l glucose (2.2 and 2.9 mmol/l/min, respectively) and report a 10 times lower glucose uptake in comparison to previous studies on isolated rat islets (17 and 32 mmol/l/min) [148, 152] (Fig. 1b). The authors stated that the low glucose uptake velocity of human islet cells was more compatible with a low Km transporter such as GLUT1 or GLUT3 [152]. However, it must be noted that these experiments were performed with dissociated human islets that contained approximately 52% β-cells; hence, the author’s conclusion on glucose transport in β-cells needs to be considered cautiously [152]. Perifusion experiments revealed a left-shifted GSIS profile of human islets compared to mouse islets [1, 114]. In comparison to their murine counterparts, human islets secreted more insulin at low glucose concentrations (5.4 mmol/l) but showed a decreased stimulation index at high glucose levels (16.7 mmol/l) [22, 114] (Fig. 2c). Glucokinase activity is similar in islets of both species while ATP concentration in human islets rose at lower glucose levels (3.5 mmol/l) compared to mouse islets (5 mmol/l) [114]. The authors explained this finding with a predominant expression of the high affinity, low Km transporters GLUT1 and GLUT3 [114]. However, it should be noted that other factors, such as the specific composition of mouse and human islets as well as the different molecular equipment of β-cells might account for this effect. In the same study, selective inhibition of GLUT1 using STF-31 led to a decreased insulin response in human β-cells, while mouse islets were unaffected, which is a strong evidence for a dominant role of GLUT1 in human β-cells [114].

Ohtsubo et al. showed that next to GLUT1, also GLUT2 contributes to glucose uptake in human β-cells [101]. In siRNA-induced knockdown experiments with human islet cells, only the simultaneous knockdown of GLUT1 and GLUT2 resulted in a marked reduction in glucose transport and abolished GSIS [101]. Knockdown of either *SLC2A1* or *SLC2A2* experiments showed no marked alterations. However, it must be noticed that these observations were made under knockdown conditions with 20–30% remaining *SLC2A1* and *SLC2A2* transcripts [101]. A complete knockout of either GLUT1 or GLUT2 in human islet cells might result in a different outcome. Furthermore, a possible compensation mechanism in single knockdown experiments was not examined. Nevertheless, these findings suggest that both, GLUT1 and GLUT2 contribute to glucose uptake in human β-cells.

Recent studies suggested the expression of other GLUTs in the islet of Langerhans. *SLC2A9* transcripts were identified in human islets and GLUT9 proteins were allocated to human β-cells by immunodetection [31, 48]. Expression of GLUT4, an insulin-regulated low affinity glucose transporter (Km ~ 12 mmol/l) [161] was detected in human and rat islets on mRNA and protein level as well as in the murine β-cell-derived β-TC cell line [12, 13, 64]. *SLC2A10*, encoding for GLUT10 was reported to be associated with Type 2 *diabetes* (T2D) and *SLC2A10* transcripts were found in human islets [48, 83]. GLUT11-C, an alternative splice form of the *SLC2A11* gene product that misses a homologous counterpart in rodents was detected in the human pancreas [130, 165]. In a transcriptome-wide analysis of human islet cells, expression of *SLC2A1*, *SLC2A2*, *SLC2A3*, *SLC2A6*, *SLC2A8*, *SLC2A11*, and *SLC2A13* was detected in β-cells [133].

Many present studies argue that GLUT1 and GLUT3 are the main glucose transporters in human β-cells. Accordingly, GLUT2 seems to contribute to glucose uptake in human β-cells, but does not cover a dominant role as this is the case in rodents. However, the present understanding of GLUT transport characteristics provide no explanation about how elevations in blood glucose concentrations are registered by GLUT1/GLUT3-mediated glucose uptake. According to their low Km values, GLUT1 and GLUT3 reach their maximum glucose transport velocity already at basal glucose levels in humans (3.9–6.1 mmol/l). In theory an elevation of blood glucose concentrations would not lead to an increased glucose uptake by GLUT1 and GLUT3. Provided a dominant role of GLUT1 and GLUT3 in β-cells, it is unclear how the cells are able to adapt insulin release to alternating glucose concentrations when glucose uptake via GLUT1 and GLUT3 does not change. According to our current knowledge, the adaption of glucose uptake to changes at high glucose levels (5–20 mM) requires the participation of low affinity transporters like GLUT2. Contrary, it was shown that GLUT1 can restore GSIS when expressed in GLUT2 deficient mice in vivo [147], suggesting a discrepancy between our current theoretical understanding of GLUT-mediated glucose transport as well as β-cell physiology and the processes happening in vivo. A possible solution for this contradiction is the presence of additional GLUTs with a low affinity for glucose, such as GLUT4 that contribute to the glucose uptake of β-cells and allow for an increased glucose uptake at glucose concentration higher than 5 mmol/l. Moreover, traceable glucose analogs exhibit differential transport characteristics compared to glucose. Measurements of transport velocity or glucose utilization with glucose analogs does not display the in vivo situation and might provide incorrect results and conclusions.

## Regulation of GLUT expression and function

### Cell type-specific GLUT2 regulation in murine β-cells

**Fig. 3:**
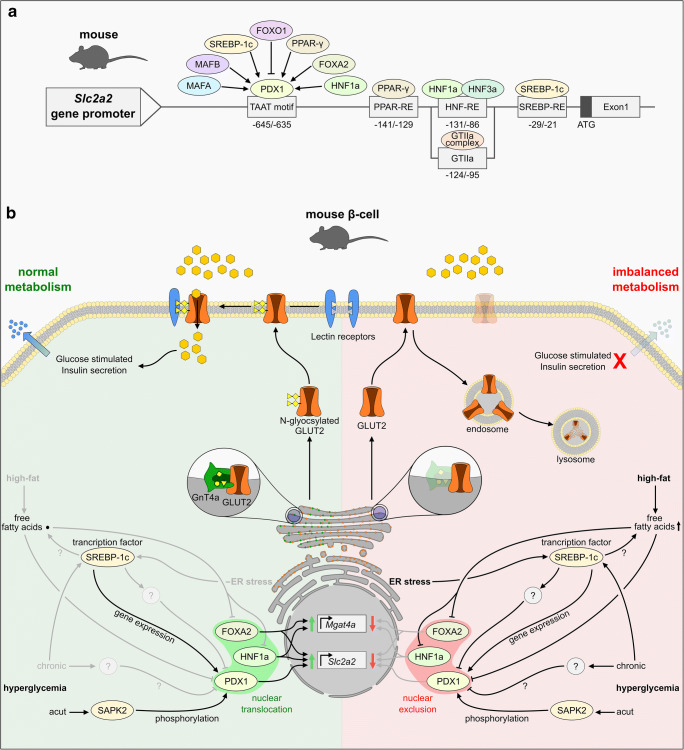
Trancriptional and poststrancriptional regulation of GLUT2 in mouse β-cells under normal and altered metabolic circumstances. **a** Overview of transcription factors with known regulatory function on *Slc2a2* gene expression in mouse β-cells and their corresponding binding sites within the *Slc2a2* promoter. In addition, factors that indirecrtly regulate *Slc2a2* expression by altering the transcriptional activity of PDX1 are shown. **b** Summary of factors and pathways affecting GLUT2 regulation and function in a normal metabolic environment (green background) and under altered metabolic conditions (red background). According to a proposed model by Ohtsubo et al. [100, 101], high amounts of free fatty acids lead to the nuclear exclusion of the transcription factors FOXA2 and HNF1a, resulting in a decreased expression of *Slc2a2* and *Mgat4a*, encoding the glycosyltransferase Gnt-4a. Consequently, N-glyocosylation of GLUT2 is impaired preventing the binding of lectin-receptors and the stabilization of GLUT2 at the cell surface. Non-glycosylated GLUT2 is increasingly found in endo- and lysosomes, resulting in a diminished glucose uptake and an impaired GSIS. Note that SREBP-1c acts as a transcriptional regulator in the nucleus and is only displayed in the cytosplasm for reasons of a clearer presentation

Transcriptional regulation of *Slc2a2* in rodent β-cells is suggested to involve several transcription factors, which directly or indirectly affect *Slc2a2* expression, including FOXA2, MAFB, MAFA, FOXO1, and PPAR-γ as well as HNF1a, HNF3a, and HNF4 [3, 4, 14, 16, 32, 55, 70, 136, 139, 168] (Fig. 3a). Tissue-specific *Slc2a2* regulation is under control of the GTIIa complex and the pancreas-specific transcription factor PDX1. The GTIIa complex binds to the cis regulatory element GTII, whereas PDX recognizes a repeat of TAAT motifs located in the promoter region of *Slc2a2* [10, 11, 77, 154, 155]. Of most importance for β-cell-specific *Slc2a2* expression is PDX1, which is known as a master regulator for genetic programs orchestrating pancreatic development and maturation of endocrine cell types [59, 95]. Early expression of PDX1 during embryogenesis correlates to the appearance of GLUT2 in pancreatic progenitor cells, underlining PDX1-controlled *Slc2a2* regulation [107, 134]. PDX1 is further involved in the transcriptional regulation of insulin and glucokinase, and thus emerges as a main transcriptional regulator of proteins involved in GSIS [72, 86, 95, 153, 158].

### GLUT2 is regulated in a glucose-dependent manner

*Slc2a2* expression in the rodent β-cell was demonstrated to be regulated in a fast, glucose-dependent manner [17]. Isolated rat islets cultured at low and high glucose concentrations showed reduced or elevated *Slc2a2* transcripts compared to islets cultured under normal glucose conditions, respectively [116, 142, 166]. Replacement of glucose by metabolized sugars (mannose and glyceraldehyde) mimicked this effect, proving that signals for transcriptional regulation of *Slc2a2* originate from glucose metabolism and not from a substrate interaction with the transporter [37].

Similar to *Slc2a2* expression, PDX1 activation is modulated according to glucose levels, and thus represents a possible mediator for glucose-dependent *Slc2a2* expression [78, 111]. In a study by Macfarlane et al., glucose metabolism stimulated stress-activated protein kinase 2 (SAPK2), which led to PDX1 phosphorylation [79]. Phosphorylated PDX1 translocated to the nucleus where it bound to its recognition sites and regulated gene expression [79, 118]. In isolated rat islets, PDX1 binding activity was decreased at low levels of glucose, while culture in high glucose preserved PDX1 binding activity, proving the glucose-dependent activation of PDX1 [78]. In mice glucose-dependent effects on *Pdx1* and *Slc2a2 expression* were abolished by the knockout of the sterol regulatory element binding protein 1c (SREBP-1c), a main player in the synthesis of triglycerides, suggesting a functional lipid metabolism as a prerequisite for the adjustment of GLUT2 expression in accordance with varying glucose levels [26]. In contrast, activation of SREBP-1c in rat islets caused by chronic hyperglycemia and endoplasmatic reticulum stress resulted in lipid accumulation and led to the downregulation of *Pdx1*, indicating that a balanced metabolism is key for *Pdx1* and *Slc2a2* expression as a requirement for β-cell functionality [157].

Contrary to the enhanced activity of PDX1 upon acute glucose increase, chronic exposure to hyperglycemia resulted in the downregulation of PDX1 mRNA and protein in HIT-15 cells, an insulin reporter-expressing cell line derived from hamster islet cells [102, 103]. Olson et al. hypothesized that downregulation of PDX1 and its target genes, including *Slc2a2*, in response to chronic hyperglycemia represented a protective event to prevent β-cells from glucotoxic effects [102, 103]. Whether the conclusion drawn from this cell line experiments applies also for more sophisticated experimental models needs to be proven.

### Bad fat—GLUT2 regulation is connected to the lipid metabolism

Nutrient-dependent transcriptional regulation of *Slc2a2* comprises not only glucose but also lipid metabolism. Rats fed a high-fat diet exhibited reduced expression of *Slc2a2* alongside with elevated blood glucose and decreased plasma insulin levels [63]. The reduction in *Slc2a2* transcripts can be explained by either chronic hyperglycemia resulting from the high-fat diet or a direct fat-dependent regulation of *Slc2a2* expression. Indeed, isolated rat islets exposed to elevated levels of fatty acids showed similarly reduced *Slc2a2* expression ratios accompanied by reduced *Pdx1* transcript levels and a decreased binding activity of PDX1 to the *Slc2a2* promoter, thus demonstrating a PDX1-mediated lipid metabolism-dependent control of *Slc2a2* expression [43]. Interestingly, mice fed a high-fat diet lost glycemic control after 1 week before a reduction in *Pdx1* and *Slc2a2* transcript levels was detectable after 4 and 8 weeks, suggesting high dietary fat as a cause for the deregulation of the PDX1 transcription axis [119].

Besides transcriptional control of *Slc2a2*, GLUT2 activity is also regulated on the posttranslational level (Fig. 3b). Ohtsubo et al. revealed correct N-glycosylation by the GnT-4a glycosyltransferase as a prerequisite for proper localization of GLUT2 at the cell surface [100, 101]. Loss of glycosylation in GnT-4a-deficient mice attenuated GLUT2 cell-surface half-life and induced endosomal and lysosomal accumulation, which eventually resulted in β-cell dysfunction similar to T2D [100]. Interestingly, similar effects were observed in mice fed a high-fat diet [100, 101]. Elaboration on the underlying mechanisms showed that high-fat nutrition led to a nuclear exclusion of the transcription factors HNF1a and FOXA2 in β-cells, causing a diminished transcription of the GnT-4a encoding *Mgat4a* gene [101]. Mice on high fat diet exhibited decreased GLUT2 surface expression, hyperglycemia, impaired glucose tolerance and hyperinsulinemia [101]. Human islets from T2D donors showed a similar pathological pattern, indicating that this pathway constitutes a relevant mechanism in the development of T2D [101].

The complexity of GLUT2 regulation was extended by studies that suggest a circadian- and age-dependent regulation of GLUT2 [39, 92]. In Goto Kakizaki rats, an inbred diabetic rat model displaying hyperglycemia and an increased glucose intolerance, aging resulted in a progressive decrease of *Slc2a2* expression, an accumulation of cytoplasmic GLUT2 protein and impaired GSIS [39]. The possibility that the circadian rhythm impacts on *Slc2a2* expression and GLUT2 function might be a considerable factor for future experimental setups, as a circadian regulation could account for the recently reported heterogeneous expression profiles of glucose transporters among individuals [128].

Interestingly, Thorens et al. described downregulation of GLUT2 expression when healthy islets were transplanted into diabetic mouse models (*db/db* or STZ-induced) [145]. Transfer of islets from *db/db* mice into mice with a non-diabetic phenotype (*db*/+) reversed this effect, suggesting that the diabetic environment consolidates the loss of β-cell function [145]. The factors inducing the reduction of GLUT2 as well as the mechanism of downregulation remain elusive. Also, it is not known at which time point in the chain of events during the onset and manifestation of *diabetes mellitus* GLUT2 downregulation takes place. Examination of the diabetic environment in the era of exosome, microRNA, and multi-omics analysis may be informative in identifying external factors affecting GLUT2 expression.

As suggested by many studies, transcription, translation, and localization of GLUT2 seem to be regulated in a nutrient-dependent manner encompassing lipid and glucose metabolism, and imbalance of both is associated with the downregulation of GLUT2 glucose uptake activity (Fig. 3b). Whether the dysregulation of GLUT2 alone is enough to account for the accompanying diabetic symptoms or is an accompanying event in an overall impaired β-cell physiology is an open question.

### Hints that GLUT1 is regulated in a nutrient-dependent manner

A recent study suggested that *Slc2a1* is regulated in a nutrient-dependent manner in mouse islets [141]. High fat, high-sucrose fed C57BL/6 as well as *db/db* mice exhibited lower levels of *Slc2a1* transcripts than control mice, which was accompanied by an enhanced secretion of glucagon [141]. Phloretin treatment of isolated mouse islets led to a reduction of *Slc2a1* expression and an increased glucagon secretion, indicating a potential direct link between GLUT1-mediated glucose uptake and the regulation of glucagon release [141].

Due to its low abundance in mouse β-cells, less information about the tissue-specific expression of GLUT1 is available. However, reports about GLUT1 regulation in other tissues provide an idea as to how GLUT1 might be regulated in β-cells. In erythrocytes, GLUT1 transport activity is directly modulated by ATP [8, 15, 73, 74]. ATP binding to the adenylate kinase homology domain 3 within the GLUT1 protein (AKHD3, GLUT1 residues 332–343) induces a conformational change resulting in an altered transport affinity for glucose and a reduced net sugar uptake [8, 15, 73, 74]. ATP-binding is competitively inhibited by AMP and ADP allowing a short-term adaption of GLUT1 activity [50].

This mechanism of ATP-controlled glucose uptake might be of great interest for the regulation of glucose uptake in human β-cells as well as other endocrine cell types. A direct short-term regulation for GLUT2 is unknown, meaning that the regulation of glucose transport in human β-cells might occurs differently than what is assumed so far. However, an ATP-dependent regulation of GLUT1 in β-cells has not been reported in literature and needs to be proven in future.

## GLUT expression and function in the diabetic disease

Impairment of the endocrine system results in the loss of glycemic control, which in case of hyperglycemia is described as *diabetes mellitus.* Causes leading to the development of *diabetes mellitus* are manifold, but eventually result in insulin resistance of peripheral cells or β-cell failure, which manifest in a diminished glucose tolerance and a reduced GSIS. Patients with a relative lack of insulin are not insulin-dependent, as β-cells are able to compensate for the insulin resistance with an increased hormone release, which is typically the case in early phases of T2D. However, abolished insulin secretion due to subsequent β-cell death leads to an absolute lack of insulin demanding insulin therapy. Type 1 diabetes (T1D) patients suffer from an absolute lack of insulin as a result of an autoimmune response towards β-cells.

An involvement of GLUTs in the disease onset or progression of both T1D and T2D has been discussed. Whereas a coherency of GLUTs and T1D appears rather unlikely, clinical and animal studies provide evidence for a correlation of GLUT impairment and T2D.

### GLUT2 expression in rodent diabetes models

Many diabetic models displaying symptoms of human *diabetes mellitus* are available*.* Interestingly, many of these models exhibit an altered GLUT2 expression and suggest GLUT2 to cover a role in the onset and manifestation of T2D (Table 2).

**Table 2:** Overview of rodent *diabetes* models with reported GLUT2 expression

Model	Obesity	GLUT2 expression	Reference
Goko-Kakizaki (GK) rats	No	GLUT2^+^ β-cells decreased	[81, 96]
		Surface GLUT2 decreased	
		mRNA decreased	
		3-OMG uptake reduced	
Diabetic (db/db) mice	Yes	GLUT2^+^ β-cells decreased	[11, 146]
		Protein decreased	
		mRNA decreased	
Zucker Diabetic Fatty (ZDF) rats	Yes	mRNA decreased	[58, 98, 99, 104]
		Protein decreased	
		GLUT2^+^ β-cells decreased	
		3-OMG uptake reduced	
STZ-induced rats	No	Protein decreased	[18, 145]
STZ-induced mice	No	mRNA decreased	[156]
		Protein decreased	
Dexamethasone-Induced (in Wistar and Zucker fatty rats)	No	GLUT2^+^ β-cells decreased^WD + ZD^	[93, 97]
		mRNA unaltered^WD^ or elevated^ZD^	
		3-OMG uptake reduced^ZD^	
DIO C57BL/6 mice	Yes	Protein decreased	[119]
Pancreatectomy in Sprague-Dawley rats	No	mRNA decreased	[167]
		Protein decreased	
BB/W rats	No	Protein decreased	[105]
		GLUT2^+^ β-cells decreased	

Obese rodent models, such as the Zucker diabetic fatty (ZDF) rat or *db/db* mice are widely used as non-insulin-dependent *diabetes mellitus* models displaying hyperglycemia as a result of insulin resistance, thereby resembling early phases of human T2D. In obese T2D models, a general decrease in GLUT2 expression is detectable, which is accompanied by a reduced GSIS.

The ZDF *diabetes* rat model was established by repeated inbreeding of fatty Zucker rats with spontaneously developed hyperglycemia and glucose intolerance [112, 170]. Male ZDF rats develop hyperglycemia between 8 and 10 weeks of age, while female rats retain glycemic control [170]. In two studies, the degree of hyperglycemia correlated with an increased loss of GLUT2-expressing β-cells, a decline in GLUT2 mRNA and protein along with a reduced β-cell mass resulting in a diminished uptake of 3-OMG and impaired GSIS in pancreases isolated from ZDF rats [58, 104]. Extended analyses revealed that reduced GSIS was due to defects at multiple sites of glucose metabolism, indicating that GLUT2 downregulation is one of many events in the dysregulation of β-cells in these *diabetes* models [98]. GLUT2 reduction as well as β-cell depletion is suggested to be promoted by excessive caloric intake, as such defects were prevented by caloric restriction [99].

Similarly, *db/db* mice, which are insulin resistant due to a mutation in the leptin receptor, exhibited a marked reduction in GLUT2^+^ β-cells and a diminished GSIS [2, 146]. Bonny et al. reported a correlation between reduction of GLUT2 mRNA and protein expression and decreased DNA binding activity of GTIIa, suggesting GTIIa to be involved in the progression of β-cell dysfunction of *db/db* mice [11].

In contrast to obese models, lean diabetic models do not show insulin resistance, but an absolute lack of insulin due to defective β-cell function. The decrease of GLUT2 is a common observation similar to obese models. Goto-Kakizaki (GK) rats were established by repeated inbreeding of glucose intolerant rats and display hyperglycemia as a result of diminished insulin release from β-cells [106, 115]. In this model, a gradual decrease in GLUT2 surface expression as well as the total amount of GLUT2^+^ β-cells and 3-OMG uptake was observed. Accordingly, rats displayed an increased insensitivity to glucose [96]. However, whether GLUT2 reduction alone caused the profound reduction of GSIS was questioned by the authors [96].

Rats and mice treated with STZ, a β-cell-specific toxin develop *diabetes* as a result of β-cell ablation. Studies reported decreased GLUT2 mRNA and protein expression correlating to the amount of STZ injections [145, 156]. STZ treatment resulted in a heterogeneous population of mice with varying severity of hyperglycemia, suggesting a diverging sensitivity of individual subjects to STZ toxicity [145]. The decrease of GLUT2 expression correlated with the extent of hyperglycemia. Due to the gradual loss of GLUT2 with increased numbers of STZ injections, GLUT2 was postulated as a direct target of STZ [156].

### Marked GLUT2 reduction induces diabetic symptoms

**Fig. 4:**
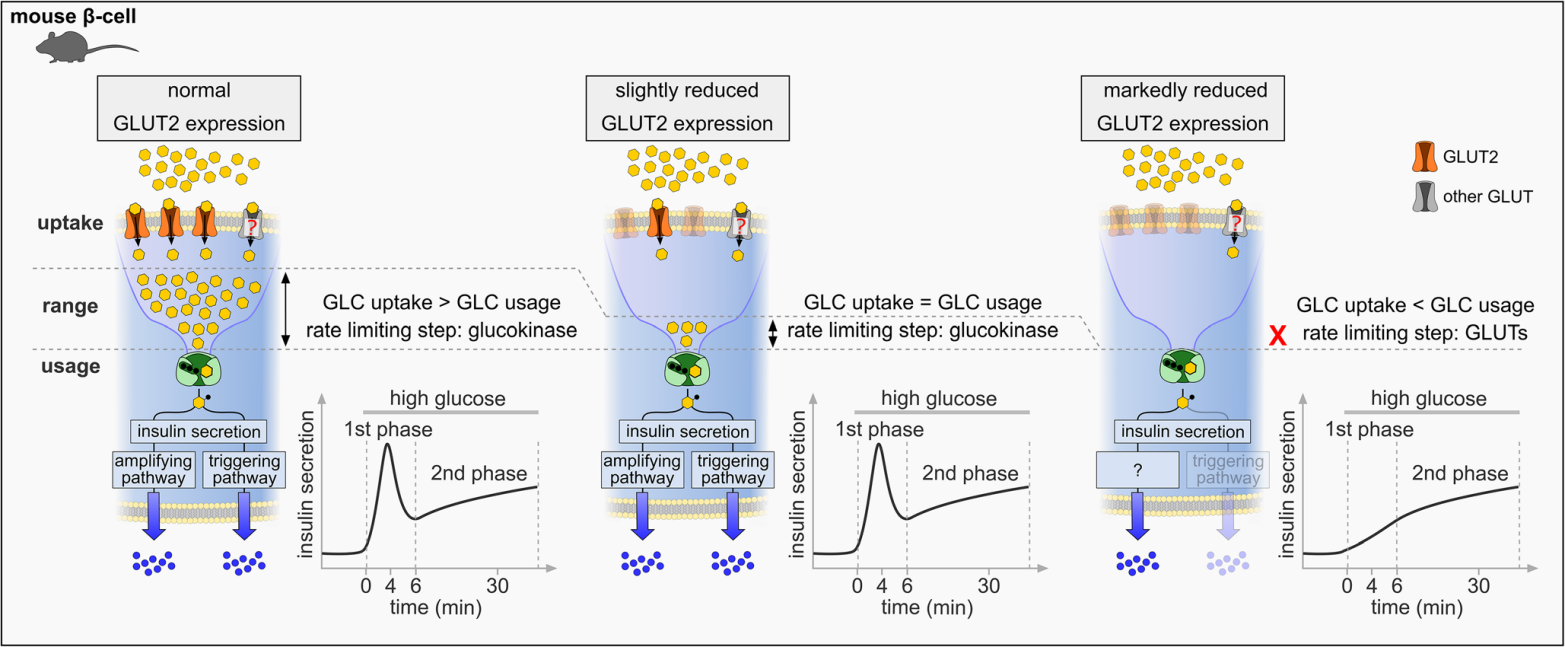
Physiological mechanism of a proposed concept for GLUT2-induced diabetic symptoms. Under normal conditions (left), glucose uptake by far exceeds subsequent glucose phosphorylation, making glucokinase the rate-limiting step in GSIS. Accordingly, both, triggering and amplifying pathway are activated resulting in a biphasic GSIS. A slight reduction in GLUT2 surface expression (middle) has no impact on GSIS, as long as glucose uptake is ≥ glucose usage. When GLUT2 is markedly reduced (right) glucose uptake falls below the rate of glucose usage, making glucose uptake the rate-limiting step in GSIS. Consequently, the triggering pathway cannot be activated, resulting in a monopahsic GSIS. The contribution of additional GLUTs (gray) to the sustained second phase of GSIS is unclear. The mechanisms underlying retained insulin secretion are unknown

Observations from diabetic animal models raise the question whether the overt GLUT2 decrease is a concomitant of *diabetes mellitus* or drives the progression of the disease. Studies prove that GLUT2 deficiency can induce *diabetes* [46, 149] (Fig. 4). GLUT2-null mice and mice expressing *Slc2a2* siRNA in β-cells displayed all the major traits of human *diabetes mellitus* [46, 149]. GLUT2 knockout mice (*Slc2a2*^*−/−*^) exhibited an impaired GSIS with diminished glucose clearance and insulin secretion upon glucose stimuli, which progressed over time and led to the death of the mice 2–3 weeks after weaning [46]. In particular, first phase insulin secretion was abolished, while second phase insulin secretion was preserved, proving that the low affinity uptake of glucose via GLUT2 is essential for fast insulin responses in rodents [46].

Studies with a *Slc2a1* siRNA-expressing mouse model exhibiting an 80% depletion of GLUT2 correlate similar findings [149]. GLUT2-deficient mice failed to adapt insulin secretion to high glucose concentrations (11.1 and 16.7 mmol/l), while hormone responses to low glucose concentrations (0 and 2.8 mmol/l) remained unaffected [149]. This is in line with studies, which demonstrated that marked reduction of GLUT2 led to a delayed insulin release upon hyperglycemic stimuli [35]. Importantly, impairment of GSIS was only detected in mouse models with a severe reduction in GLUT2 or homozygous knockout mice, but not in heterozygous animals [35, 46, 149]. This can be explained by the transport characteristics of GLUT2. Under normal conditions, glucose uptake by GLUT2 by far exceeds glucose phosphorylation via glucokinase, resulting in a physiological range at which reduction of GLUT2 expression is without consequences for insulin response [142] (Fig. 4). However, a marked decrease of GLUT2 can reverse this relation, making glucose uptake the rate-limiting step in GSIS. Thus, under experimental conditions, chronic decrease of GLUT2 activity can result in a reduction of β-cell mass and the onset of diabetic symptoms.

### GLUTs in human T2D islets

Similar to rodent diabetic models, a reduction of certain GLUTs is observed in human T2D islets [23, 101]. Guerra et al. described a reduced gene expression of *SLC2A1*, *SLC2A2*, and *GCK* (encoding glucokinase) in human T2D islets exhibiting a slight reduction in β-cell mass (~ 10%) [23]. Downregulation of GLUT1 and GLUT2 was accompanied by reduced glucose utilization and decreased insulin secretion at 16.7 mmol/l glucose compared to healthy islets [23]. Guerra et al. further observed an elevated amount of *PDX1* and *FOXO1* transcripts [23]. As shown in rodent models, the transcription activity of PDX1 requires phosphorylation, which is linked to lipid and glucose metabolism [43, 78, 111]. Unfortunately, the study does not provide data about the binding activity of PDX1 to the *SLC2A1* and *SLC2A2* promoters [23]. Furthermore, the simultaneous increased expression of *PDX1* and *FOXO1*, an inhibitor of *PDX1* expression, is contradictory. It might be that the observed elevation of *PDX1* expression represented a compensatory effect for counterbalancing a diminished PDX1 promoter binding activity and a general β-cell dysfunction.

Equally to their observations made in mice fed a high-fat diet [100], Ohtsubo et al. reported nuclear exclusion of FOXA2 and HNFA in > 70% of islet cells from two T2D patients, while both transcription factors were predominantly localized in the nucleus in cells from healthy donors [101]. This observation was accompanied by a reduced expression of *SLC2A1* and *SLC2A2*, as well as *MGAT4A*, encoding for GnT-4a glycosyltransferase [101]. T2D islets further exhibited 80–90% reduced surface expression of GLUT1 and GLUT2 as well as diminished glucose uptake and lacked GSIS [101]. Ohtsubo et al. were able to establish a similar phenotype in healthy islets by administrating palmitic acid and thereby unraveled a potential pathway linking lipotoxic effects with impaired GSIS [101]. According to their proposed model, administration of palmitic acid to the culture of isolated human islets led to the nuclear exclusion of FOXA2 and HNFA in β-cells, which resulted in a reduced expression of *SLC2A1* and *SLC2A2* as well *as MGAT4A* [101]*.* Downregulation of *MGAT4A* translated into a reduced GnT-4a activity resulting in an insufficient glycosylation of β-cell proteins, including GLUT1 and GLUT2 [101]. Missing glycosylation prevented binding of GLUTs to lectin receptors, which led to an attenuated surface half-life of GLUT1 and GLUT2 [101]. Consequently, glucose uptake in β-cells was diminished. Whether the observed impairment of GSIS was solely due to the decreased surface half-life of GLUT1 and GLUT2 or was caused by the reduced expression of other HNF1A-controlled genes that are involved in GSIS is not answered by this study.

Overall, studies on rodent *diabetes* models and human T2D islets suggest a coherence between GLUT1 and GLUT2 decrease and the development of T2D. Under experimental conditions, manipulated GLUT expression is shown to induce *diabetes*, and thus presents one of many putative ways how *diabetes mellitus* can be triggered. Ohtsubo et al. linked GLUT reduction to an enhanced amount of free fatty acids, which is a common feature of prediabetic T2D patients, thereby providing a model for nutrient-dependent dysregulation of GLUT1 and GLUT2 in the course of *diabetes* in mice and humans [101]. However, whether the downregulation of GLUT1 and/or GLUT2 presents an initial event in pathological pathways causing *diabetes* needs to be proven.

### The role of GLUTs in the development of T1D

T1D results from a loss of mature β-cells due to an autoimmune reaction, causing an absolute lack of insulin. The few studies that investigated the involvement of GLUTs in this event provide conflicting data.

Biobreeding (BB) rats are an inbred strain that spontaneously develop autoimmune insulitis, resulting in loss of β-cell function closely resembling human T1D [90]. A study investigating GLUT2 expression in BB rats described a reduction of GLUT2^+^ β-cells proceeding the onset of T1D [105]. Thereby, rats with overt *diabetes* exhibited less GLUT2^+^ β-cells than pre-diabetic mice and both cohorts revealed a reduced amount of GLUT2^+^ β-cells compared to control mice [105]. Loss of GLUT2 correlated to a temporal reduction in GSIS that preceded T1D onset [105].

In contrast to these studies, *diabetes*-resistant BB/Wor rats, treated with Kilham Rat virus to induce T1D did not show any alteration in the amount of GLUT2^+^ β-cells in the initial stage after virus infection [140]. A decrease in GLUT2^+^ β-cell mass became obvious with the onset of insulitis, arguing that GLUT2 is not essentially involved in the etiology of Kilham Rat virus-induced T1D [140].

Furthermore, an extensive study from Coppitiers et al. demonstrated the persistent expression of GLUT1 in islets from human patients, even after longstanding T1D [20]. Similarly, in mice, GLUT2 expression was conserved in trace quantities in few remaining insulin-expressing cells, even in the course of inflammatory stress [20]. GLUT2^−^/insulin^+^ β cells were found in areas with direct contact to the site of inflammation [20].

Other studies suggested the presence of autoantibodies against GLUT2 in the sera of T1D patients [56, 109]. Using GLUT2- or GLUT1-expressing AtT-20INS cells cultured with purified IgG sera of patients with recent onset of T1D, a specific binding of autoantibodies to GLUT2 could be demonstrated, which led to a reduced transport of 3-OMG [56]. So far, the findings obtained in this highly artificial experimental setup, were not confirmed in isolated islets or in vivo.

The knowledge from clinical observations as well as animal models do not provide a clear picture about the role of GLUT1 and GLUT2 in T1D. Findings are highly contradictory and remain speculative.

### Clinical pictures of human GLUT1 and GLUT2 mutations

Other than in rodents, impaired GLUT2 function in humans is only rarely associated with *diabetes* [124]. Patients carrying biallelic mutations in the *SLC2A2* gene display disorders in renal and hepatic carbohydrate metabolism, known as Fanconi Bickel syndrome (OMIM #227810) [34, 125]. Although, postprandial hyperglycemia and glucose intolerance in these patients indicate a dysregulation of β-cells, overt *diabetes* is rarely observed and the associated symptoms are not comparable with the severe complications of GLUT2-deficiency in rodent models [126, 127]. There are reports about transient neonatal *diabetes* occurring in patients with a homozygous *SLC2A2* mutation [61, 122, 124]. In a study comprising 104 patients with diagnosed transient (*n* = 25) or permanent (*n* = 79) neonatal *diabetes*, 5 patients were found to carry a homozygous *SLC2A2* mutation [124]. Of these five patients, one exhibited permanent neonatal *diabetes* that required a continued insulin therapy [124]. These data indicate a transient requirement of GLUT2 for β-cell function in infants and a switch of GLUT expression in the first month of life [47]. Furthermore, the reports about varying phenotypes between patients with the same mutation raise the question to which extent glucose transporter expression varies among individuals [40]. Recent transcriptome analysis of human pancreatic islets from 207 donors provide evidence for a huge inter-individual heterogeneity for glucose transporters expression profiles [128].

According to the assumption that GLUT1 presents the main glucose transporter in human islets, it is expected that the knockout of GLUT1 results in severe glycemic implications. However, the clinical picture of patients with mutations in the *SLC2A1* gene, known as glucose transporter 1 deficiency syndrome (Glut1DS, OMIM #606777) comprise neurological disorders, but not an impairment of glycemic control [49, 71].

Concerning the endocrine function, mutational defects of the putative main glucose transporters in the human islet, GLUT1 and GLUT2 remain without clinical symptoms. This raises the questions, whether other transporters are involved in glucose uptake of human β-cells or if other mechanisms can compensate for the loss of single transporters.

## Sodium-glucose cotransporters in the pancreatic islet

Among the protein family of sodium-glucose cotransporters, SGLT1 and SGLT2 characterize the two best studied members simultaneously transporting glucose and sodium (Na^+^) across cell membranes against its concentration gradient using the electrochemical force generated by a sodium-potassium (Na^+^-K^+^)-ATPase [21, 163, 164]. SGLT1 is predominantly expressed in the small intestine, playing the leading role for dietary glucose uptake [42, 123]. In addition, it transports a small amount of filtered hepatic glucose in the late proximal tubule of the kidney, while the bulk of filtered hepatic glucose is reabsorbed in the early proximal tubules by SGLT2 [60, 121, 163, 164]. In view of these classical physiological roles, SGLT1 and SGLT2 are of high importance for blood glucose homeostasis. Given their recent identification also in organs other than the small intestine and kidney, this review will summarize current knowledge about individual expression profiles (Table 3) and suggested functional roles for SGLT1 and SGLT2 in the human and rodent pancreatic islet.

**Table 3:** Overview on the distribution of SGLT1 and SGLT2 in mouse, rat, and human pancreatic cells as well as pancreatic cell lines

SGLT isoform	Species	Localization	Details and reference
SGLT1	Mouse	Islet α-cells	SGLT1 mRNA and protein expression [9].
			SGLT1 protein expression [141].
			SGLT1 mRNA expression [137].
		Islet β-cells	Low SGLT1 mRNA expression levels [66].
		Islet δ-cells	Low SGLT1 mRNA expression levels [66].
		Islet cluster	Increased SGLT1 mRNA levels in islets isolated from *ob/ob* mice followed by a decline of expression levels in aged *ob/ob* mice [9].
			Low SGLT1 mRNA expression [66].
			SGLT1 mRNA and protein expression [141].
			Increased SGLT1 expression levels in *db/db* mice reported [141].
			Low SGLT1 mRNA expression levels [89].
		α-TC1 cell line	SGLT1 mRNA expression [137, 141].
			Increased SGLT1 expression levels at high glucose conditions reported [141].
		Pancreas in total	Low SGLT1 mRNA expression levels [80].
		Exocrine pancreas	SGLT1 protein expression in duct cells [80].
			Low SGLT1 mRNA expression levels [89].
	Human	Islet α-cells	SGLT1 mRNA and protein expression [9].
			SGLT1 mRNA expression [27, 66, 133].
		Islet β-cells	Low SGLT1 mRNA expression levels [66].
		Islet cluster	Enriched SGLT1 mRNA levels in islets isolated from obese, glucose intolerant or T2D patients compared to lean controls reported [9].
			In contrast to SGLT1 in the murine islet and the findings observed for SGLT2, SGLT1 mRNA ratios remained elevated during the progression from insulin resistance to diabetes shown [9].
			SGLT1 mRNA and protein expression [137, 141].
			High variability of SGLT1 expression in human islet samples isolated from different donors. In average, SGLT1 mRNA transcripts are more abundant than SGLT2 mRNA transcripts reported [128].
	Rat	Islet cluster	Low levels SGLT1 mRNA expression [66].
		Pancreas in total	Low SGLT1 mRNA expression levels [80].
SGLT2	Mouse	Islet α-cells	SGLT2 protein expression [9].
			Marginal, below the detection limit, SGLT2 mRNA signals reported [66].
			Few SGLT2 transcripts [151].
		Islet β-cells	Marginal, below the detection limit, SGLT2 mRNA signals reported [66].
			Few SGLT2 transcripts [151].
		Islet δ-cells	Specific expression of SGLT2 transcripts in 58% of δ-cell population reported [151].
			Marginal, below the detection limit, SGLT2 mRNA signals reported [66].
		α-TC1 cell line	SGLT2 mRNA and protein expression [62].
			Temporary high glucose concentrations resulted in an increase in SGLT2 mRNA and protein expression in murine α-TC1 cells. Culture up to 72 h at high glucose concentrations resulted in a decrease in SGLT2 mRNA and protein expression in murine α-TC1 cells reported [62].
		Islet cluster	SGLT2 mRNA expression [9].
			SGLT2 protein expression [9].
			SGLT2 expression increased in *ob/ob* mice from 5 to 8 weeks and declined at the age of 15 weeks when mice became overtly hyperglycemic reported [9].
	Human	Islet α-cells	SGLT2 mRNA and protein expression [9].
			Enriched mRNA levels in islets isolated from obese, glucose intolerant or T2D patients compared to lean controls [9].
			Marginal, below the detection limit, SGLT2 mRNA signals reported [66].
			Few SGLT2 transcripts [151].
		Islet β-cells	Few SGLT2 transcripts [151].
			Marginal, below the detection limit, SGLT2 mRNA signals reported [66].
		Islet δ-cells	Specific expression of SGLT2 transcripts in 33% of δ-cell population reported [151].
			Marginal, below the detection limit, SGLT2 mRNA signals reported [66].
		Islet cluster	Marginal, below the detection limit, SGLT2 mRNA signals reported [66].
			SGLT2 mRNA expression [128].
			SGLT2 mRNA expression in human donors is highly heterogeneous [128].
			Pearson’s correlation underlines SGLT2 and glucagon colocalization by [128].
		Pancreas in total	SGLT2 mRNA expression [19].

### SGLT2 expression and suggested functional roles in the pancreatic islet

Blocking renal SGLT2 activity is beneficial for glycemic control [143]. However, clinical studies reported elevated plasma glucagon levels, an enhanced endogenous glucose production (EGP) and diabetic ketoacidosis in SGLT2 inhibitor-treated diabetic patients [36, 85, 94, 110, 129]. These mechanistic side effects are unlikely to originate from blocking renal SGLT2 alone, but might be the consequence of an inhibited SGLT2 activity in other tissues, including the pancreas. In the following, we will focus on cell type-specific expression profiles of SGLT2 and its hypothesized role in the pancreatic islet. For detailed information on SGLT2 inhibitors, we refer interested readers to excellent reviews about this topic [65, 120, 143].

In 2010, the presence of SGLT2 mRNA transcripts in the human pancreas was first reported by Chen et al. [19]. Five years later, Bonner and colleagues demonstrated SGLT2 mRNA and protein expression in human glucagon-secreting α-cells, being differentially regulated under conditions of altered glucose homeostasis with enriched SGLT2 mRNA levels in obese, glucose intolerant (GI) and T2D islets compared to lean controls [9]. Gene expression analyses revealed lower SGLT2 mRNA ratios in T2D than GI islets, while mRNA expression levels for glucagon were elevated in T2D samples [9]. Hypothesizing a link between SGLT2 and glucagon, Bonner et al. demonstrated an increased glucagon secretion by human islets when SGLT2 activity was abrogated by RNAi-induced knockdown or by the treatment of islets with dapagliflozin [9], a selective SGLT2 inhibitor [159, 160]. Similar to their findings for human islets, Bonner et al. confirmed SGLT2 protein expression in murine pancreatic α-cells [9]. Islets isolated from *ob/ob* mice exhibited elevated SGLT2 mRNA levels compared to healthy controls. While SGLT2 mRNA expression ratios increased from 5 to 8 weeks after birth, a decline in mRNA levels was noticed in 15-week-old, overtly hyperglycemic *ob/ob* mice, suggesting SGLT2 expression in *ob/ob* mice to be regulated by age and/or progression of insulin resistance [9]. Interestingly, 15-week old hyperglycemic *ob/ob* mice demonstrated increased glucagon mRNA levels, similar to the elevated glucagon expression ratios observed in human T2D islets [9]. Moreover, the authors showed that SGLT2 inhibition by dapagliflozin treatment resulted in elevated plasma glucagon levels in healthy C57BL/6 mice [7], demonstrating the physiological relevance of their data. Pederson and colleagues confirmed a dapagliflozin-mediated effect on glucagon secretion in human and murine islets [108]. Their mathematical model of human α-cell electrophysiology revealed that SGLT2 contributes to the regulation of glucagon secretion by α-cells possibly via an electrical mechanism and that blocking SGLT2 activity partly attenuates the suppression of glucagon release at high glucose concentrations by allowing full action potentials to develop [108, 169]. Suggesting a regulatory role for SGLT2 in the context of glucagon secretion by human islet α-cells, the findings of both studies could partially explain the elevated plasma glucagon levels and therefore the increase in EGP observed in diabetic patients treated with current SGLT2 inhibitors [36, 85]. Earlier it was assumed that this class of agents mainly acts in the kidney to promote glucosuria thereby improving glycemic control.

The effect of SGLT2 inhibition on glucagon secretion under dynamically changing glucose concentrations ranging from low to high blood glucose levels strongly requires further investigations in the future, as these effects may be disadvantageous in diabetic patients thereby raising some concerns about current SGLT2 inhibitors. As SGLT2 inhibition seems to be linked to the regulation of glucagon release, SGLT2 inhibitor-based combination therapies with agents that reduce glucagon levels may be advantageous. In this context, Kim et al. studied the effect of troglitazone on α-cell-specific SGLT2 and the release of glucagon using the murine α-TC1 cell line as model system [62]. Troglitazone is a peroxisome proliferator-activated receptor γ (PPAR-γ) agonist that improves insulin sensitivity and glucose tolerance in obese subjects [91]. Furthermore, troglitazone was shown to increase SGLT2 protein expression and the uptake of α-methylglucoside (α-MG), a non-metabolizable SGLT-specific glucose analog [42, 44], in primary rabbit renal cell cultures [69]. In their study, Kim et al. confirmed the recently observed decreased SGLT2 expression in α-TC1 cells cultured under chronic hyperglycemic conditions, while glucagon expression was elevated [62]. Treatment with troglitazone resulted in an increased expression of SGLT2, thereby attenuating the hyperglycemia-induced boost of glucagon secretion [62]. Mechanistically, the PPAR-γ agonist-mediated effect was linked to the activation of the PI3K/Akt pathway [62]. Together, the authors showed a troglitazone-mediated improvement of glucagon dysregulation in pancreatic α-cells at high glucose concentrations by increasing the expression of SGLT2, providing a rational for a PPAR-γ agonists/SGLT2 inhibitor combinatorial therapy.

In contrast to the α-cell-specific expression profile reported before, Vergari et al. characterized 58% of murine and 33% of human δ-cells as dominant SGLT2-positive cell type in the pancreatic islet, while SGLT2 protein signals were detected in only a minority of other islet cell types, mainly α- and β-cells [151]. While confirming a role for SGLT2 in the context of glucagon secretion, the observations of Vergari and colleagues argue against a direct role for SGLT2 in the α-cell. The findings of their work demonstrate that insulin inhibits the secretion of glucagon from α-cells by a SGLT2-induced stimulation of somatostatin release from δ-cells, thereby suggesting SGLT2 inhibitors as useful additives for insulin-based therapies in diabetic patients [151].

Interested in mechanisms linking the selective SGLT2 inhibition to the deregulation of glucagon release observed in clinical studies [36, 85], Solini et al. reported about virtually undetectable mRNA signals for SGLT2 in murine and human islets as well as the murine α-TC1 cell line [137]. Moreover, the authors showed that dapagliflozin modulates glucagon secretion in an SGLT2-independent manner [137]. In line with this, Suga and colleagues did not observe significant expression levels of SGLT2 in murine or human islets [141]. Both groups suggested a relevant role for SGLT1, which is discussed in detail in the following section of this review.

Marginal SGLT2 mRNA reads below the detection limit were also reported by Kuhre et al. in mouse, rat and human islet clusters as well as FACS-sorted α-, β-, and δ-cells of the mouse [66]. In addition, analysis of publicly available RNA sequencing data of dispersed human islets revealed that SGLT2 mRNA expression was nearly absent also in human α-, β-, or δ-cells and its expression was not altered in T2D islets [66]. Using a physiologically relevant isolated perfused rat pancreas model, the authors could not confirm any effect of SGLT2 activity on the secretion of glucagon [66].

Summarizing recent findings, SGLT2 expression and therefore its role in the pancreatic islet is highly controversial. However, a recently published study by Saponaro et al. could explain at least to some extent the distinct results obtained in the past by different groups [128]. Whole islet gene expression analysis of datasets from the TIGER database [33, 150] demonstrated that SGLT2 expression is highly heterogeneous in the human pancreatic islet [128]. A similar variability of SGLT2 protein expression among donors was also observed in Western blot analysis, using islet lysates from 10 different donors [128]. In order to assess the precise localization of SGLT2 protein expression in endocrine islet subtypes, IHC analysis was performed and finally demonstrated the colocalization of SGLT2 protein signals with those observed for glucagon but not with insulin or somatostatin. Furthermore, 665 islet images from various donors were analyzed using the IMARIS Bitplane software where the correlation of voxels positive for two distinct channels is shown as Pearson’s correlation coefficient. The resulting Pearson’s correlation values for SGLT2 and glucagon quantitatively underlined that only SGLT2 and glucagon signals co-localized [128]. Moreover, islets isolated from 31 donors showed a heterogeneous glucagon releasing behavior at low-glucose conditions of 1 mmol/l correlating with the secretion of glucagon when dapagliflozin was applied to islets cultured at 6 mmol/l glucose [128]. Islets isolated from three distinct donors did not respond to low glucose conditions nor dapagliflozin treatment in this study [128]. Together, these findings suggest a heterogeneous expression profile for SGLT2 in human α-cells that corresponds to an inter-individual variability of their glucagon secreting capacity [128], an important aspect that should be considered in the future regarding SGLT2 inhibitor-based therapeutic strategies.

### SGLT1 represents a second SGLT family member expressed in the pancreatic islet

The reduction of postprandial glucose excursion in diabetic patients is a valuable strategy to improve glycemic control, which can be achieved by selective inhibition of intestinal SGLT1 or by the combined inhibition of SGLT1/2 in the small intestine and kidney. While SGLT2 inhibitors are frequently used in the clinic, the role of SGLT1 inhibition is still under investigation and the recently identified new immunolocalizations of SGLT1 represent a considerable factor in this context.

Pancreatic islet-specific SGLT1 expression was first reported by Bonner et al. in course of their SGLT2 studies described above [9]. Similar to SGLT2, SGLT1 mRNA and protein expression was observed predominantly for the α-cell population of human and murine islets [9]. Corresponding to the regulation of SGLT2, the authors also demonstrated that islet-specific SGLT1 is affected by alterations in glucose homeostasis. Compared to wild type controls, increased *Slc5a1* levels were observed in islets isolated from insulin resistant prediabetic *ob/ob* mice, followed by a decline of *Slc5a1* expression once the diabetic phenotype was established [9]. As Bonner and colleagues were further interested if islet-specific SGLT1 is also regulated under distinct conditions of altered glucose homeostasis in humans, they studied *SLC5A1* levels similar to *SLC5A2* in islets from donors with diverse health states [9]. Compared to lean controls, the authors observed elevated *SLC5A1* values in all human islets with a disease background. In contrast to SGLT1 in the murine islet and the findings observed for SGLT2, SGLT1 mRNA ratios remained elevated during the progression from insulin resistance to *diabetes* [9]. Contrarily, SGLT2 mRNA levels were lower in T2D islets compared to islets from glucose intolerant individuals [9]. Similar to SGLT2, the findings from Bonner et al. indicate a role for SGLT1 in the progression of *diabetes* in mice and humans, albeit with variances in both species. Furthermore, while SGLT1 and SGLT2 seem to be similarly regulated in murine islets with an obese phenotype, differences were observed for both transporters in human islets when insulin resistance progresses to T2D.

The islet-specific expression of SGLT1 was later confirmed by others demonstrating high SGLT1 mRNA and protein levels in murine and human pancreatic α-cells [137, 141]. The constitutive expression of SGLT1 in the murine α-TC1 cell line further supports the α-cell-specific expression profile [137, 141]. Controversial findings were made for a possible glucose-mediated regulatory effect on SGLT1 mRNA expression in murine α-TC1 cells. While Solini et al. could not observe any effect of changing glucose concentrations [137], Suga and colleagues reported that high glucose concentrations resulted in increased SGLT1 mRNA expression levels in α-TC1 cells [141]. Reasons for these differential findings remain elusive but may result from distinct experimental conditions. Nevertheless, both studies provided evidence that α-cell-specific SGLT1 is predominantly expressed over SGLT2, which was almost undetectable in murine and human pancreatic islets in both studies [137, 141]. Moreover, both groups demonstrated a correlation between SGLT1 and the regulation of glucagon release from pancreatic α-cells. Application of the SGLT2-inhibitor dapagliflozin to murine islet cultures or α-TC1 cells resulted in an increase in SGLT1 and preproglucagon mRNA expression as well as a rapid boost in glucagon release [137]. Given the virtually undetectable expression of SGLT2, the authors proposed that SGLT1 contributes to the dapagliflozin-mediated effect on glucagon release from pancreatic α-cells, which was confirmed by *SLC5A1* gene-silencing in human islets [137]. Further, they hypothesized that the dapagliflozin-mediated effect on SGLT1 and the release of glucagon may explain the elevated plasma glucagon levels reported for dapagliflozin-treated diabetic patients [36, 85, 137]. Interested in possible underlying mechanisms, an altered expression of PASK and AMPK-a2 was observed, both characterizing representative candidates of associated intracellular signaling pathways [137]. The suggested role for SGLT1 in glucagon release from pancreatic α-cells was confirmed by Suga et al., demonstrating that the siRNA-mediated knockdown of SGLT1 expression in α-TC1 cells resulted in a significantly reduced secretion of glucagon [141]. Additionally, they applied canagliflozin, a SGLT2 inhibitor with relatively weak selectivity in contrast to dapagliflozin [67, 76], which similarly led to the suppression of glucagon secretion from α-TC1 cells [141]. This was also observed for canagliflozin-treated murine islets [141]. Notably, the authors showed that canagliflozin treatment suppressed the transport of the non-metabolizable αMG glucose analog transported by SGLT1, and that this was associated with blocking the increase of intracellular Ca^2+^ levels [141]. Investigating the physiological relevance of a SGLT1-glucagon correlation, Suga and coworkers reported altered SGLT1 mRNA expression rates in islets isolated from *db/db* or HFHSD mice, the latter ones representing an obese mouse model fed a high-fat, high-sucrose diet [141]. Importantly, the higher SGLT1 mRNA levels were associated with an elevated glucagon release in *db/db* or HFHSD islets and reduced mRNA expression rates of GLUT1 [52, 141]. In summary, both studies suggest an important role of SGLT1 in regulating the release of glucagon from pancreatic α-cells, possibly together with GLUT1 as indicated by Suga and colleagues [141]. While inhibition of SGLT1 with sotagliflozin, a non-selective dual SGLT1/SGLT2 inhibitor [68] decreased the secretion of glucagon from murine islets, phloretin treatment that blocks GLUT1 activity resulted in an increase of glucagon release [141]. In view of their opposite expression patterns, the authors suggested that SGLT1 and GLUT1 may contribute to the regulation of glucagon secretion from pancreatic α-cells with opposing signals indicating a possible interplay of both transporters [141]. In view of the high SGLT1 mRNA levels under high glucose and diabetic conditions associated with an increased glucagon release from α-cells, Suga et al. suggested that an elevated SGLT1 expression and activity might represent an etiology of hyperglucagonemia in T2D [141], which needs to be further investigated in future studies. Nevertheless, to carefully determine the organ-specific role of SGLT1, precise blocking of SGLT1 activity is required using selective inhibitors in future studies.

The expression of SGLT1 in murine islets was also demonstrated by Kuhre and colleagues [66]. In addition, rat islets were shown to be SGLT1 positive [66]. Single-cell studies with sorted α-, β- and δ-cells resulted in different findings compared to previous studies, as SGLT1 was not detected in murine α-cells. Instead, Kuhre et al. reported *Slc5a1* expression in murine β- and δ-cells [66]. As already mentioned, the authors could not detect *Slc5a2* expression in murine and rat islets. In addition, they also analyzed the expression of both transporters in RNA sequencing data sets of human islets from two cohorts comprising healthy and diabetic individuals. SGLT1 transcripts were detected in human α-cells and to a lower extent in β- but not in δ-cells [66]. In addition, two other studies identified SGLT1 in islet α-cells on single-cell level in course of genome-wide transcriptome analyses [27, 133] with an enriched SGLT1 expression in α-cells compared to β-cells of non-diabetic islets [27]. Similar to SGLT2, Saponaro et al. reported a high variability of SGLT1-specific expression profiles in course of their large-scale analysis of human islet samples isolated from different donors [128].

Studies reporting about SGLT1 expression provide contradictory data. High variability of SGLT1 expression among individuals is one explanation for the heterogeneous findings [128]. A recent study provide evidence that the inconsistent findings about SGLT1 expression might originate from differences in experimental setups. Especially, in terms of investigating SGLT1 protein expression profiles, polyclonal antibodies were used in the past that were tested for their specificity in classical SGLT1-reference organs (small intestine or kidney) as well as by the combination with preabsorption of antigenic peptides. However, Madunic and colleagues questioned the specificity of lately reported SGLT1 immunoreactivity data in human and rodent organs, as the authors characterized antigenic peptides as inadequate controls due to their cross-reactivity with similar epitopes [80]. To determine the expression profile of SGLT1 in various mouse organs, Madunic and coworkers performed immunolocalization studies using a highly specific and self-made anti-mouse SGLT1 antibody. Identified signals were characterized as specific when no signal was detected in organs isolated from a global SGLT1 knockout mouse line [42]. In their study, the authors verified SGLT1 mRNA expression in the whole pancreas analyzing 3 to 5 months old C57BL6/J mice without determination of any gender-specific effect on mRNA levels [80]. mRNA profiling was performed by end-point and qRT-PCR in murine and rat organs resulting in higher mRNA expression ratios for SGLT1 in murine than in rat pancreas, which suggests diverse regulatory mechanisms or a distinct functional importance in different species. In addition, IHC analyses characterized duct cells of the exocrine pancreas as SGLT1-positive [80]. Notably, Madunic et al. could not confirm the islet-specific expression of SGLT1. Questioning these findings that differ from the recently reported α-cell-specific expression profile, the authors performed additional IHC analyses. They used commercially available SGLT1 antibodies and tested their functionality in representative SGLT1-positive organs (kidney and small intestine) concluding that the analyzed antibody variants were not suitable for the investigation of murine SGLT1 by IHC [80]. Unfortunately, the authors did not investigated SGLT1 in human pancreas. In contrast to Madunic et al., we recently demonstrated SGLT1 mRNA expression by qRT-PCR in the endocrine and exocrine pancreas isolated from C56BL6/J mice at an age of 12 weeks [89]. The absence of SGLT1 mRNA expression in age-matched negative controls isolated from the same global SGLT1 knockout mouse line as previously used by Madunic and colleagues [42, 80] confirmed these findings. In addition, we also demonstrated that SGLT1 is of high importance to maintain murine islet integrity, as the loss of SGLT1 results in enlarged islets associated with decreased proliferation and apoptosis rates [89]. Furthermore, a reduction in β-cell and an increase in α-cell mass was observed, indicating an important role for SGLT1 within the α-cell population [89]. The alterations in cellular architecture of SGLT1 knockout islets [89] may indicate a compensatory mechanism to counteract the loss of α-cell-specific SGLT1 activity in the murine islet.

## Conclusion

Much knowledge has been gathered about glucose transporters, that contributes a lot to our current understanding of glucose uptake into cells. The current literature provides clear evidence that glucose uptake by GLUTs and SGLTs covers an important part in the physiology of endocrine cells. Resolving the exact role of GLUTs and SGLTs in pancreatic cells and unraveling their contribution to cell type-specific functions remains challenging.

Due to an increasing number of clinical data and studies on human samples, there is a growing awareness that inter-species differences are greater and less understood as initially thought. This discrepancy becomes obvious in the differential expression profile of GLUT1, GLUT2 and GLUT3 in rodent and human β-cells and likewise seems to hold true for the abundance of SGLT1 and SGLT2 in the pancreatic islet. This inconsistency might depend on species-specific developmental aspects, different physiologies as well as distinct metabolic requirements among individual species and results in differential clinical pictures. Consequently, the translation of data obtained from rodent studies to humans should be done with caution.

Despite overt interspecies differences, animal models are still required as the examination of human samples is limited by their low availability. The establishment of biobanks as well as the growing amount of human pancreas datasets promise a large improvement regarding the examination and comprehension of human islet physiology in health and disease [33, 150]. Furthermore, advancing techniques such as single cell-based RNAseq and Proteomics harbor a great potential to clarify the so far conflicting picture of GLUT and SGLT expression in subtypes of the pancreatic endocrine cells. Transcriptome-wide analyses on single cell level are needed to improve our understanding of expression heterogeneity among individuals, which emerged as an important, but so far neglected aspect, during studies on SGLTs expression. Enlarging the sample size of analyzed data sets provides the possibility to obtain a comprehensive picture about glucose transporter expression in the pancreas and might help to understand the discrepancy of current studies.

Unraveling the function of GLUTs and SGLTs is further impeded by technical hurdles and overall experimental conditions comprising the use of unspecific antibodies in course of immunolocalization studies, the lack of transporter-specific inhibitors or the use of non-selective inhibitors as well as different glucose concentrations and artificial in vitro cell culture environments. A common strategy regarding sample collection, used antibodies and experimental conditions would be a large benefit for future investigations regarding GLUTs and SGLTs function in the endocrine pancreas.
